# Effect of carbon black addition on thermal stability and capacitive performances of supercapacitors

**DOI:** 10.1038/s41598-018-30507-5

**Published:** 2018-08-10

**Authors:** Kyungwhan Yang, Kyoungah Cho, Sangsig Kim

**Affiliations:** Department of Electrical Engineering, Korea University, Anam-ro 145, Sungbuk-gu, Seoul, 136-701 Korea

## Abstract

In this study, we propose a simple way to improve thermal stability of solid-state supercapacitors (SCs) by adding carbon black (CB) into reduced graphene oxide (rGO) electrodes. The CB used as a heat-resistant additive contributes to stable operation of the rGO-CB SC even after 1000 charge/discharge cycles at 90 °C. In the case of the rGO SC without CB, it fails after the 166th cycles at 90 °C. Compared with the rGO SC, the rGO-CB SC exhibits the decrease in internal resistance from 42 to 18 Ω and the increase in specific capacitance from 115 to 160 F/g. Moreover, the rGO-CB SC shows a smaller variation in specific capacitance (12%) than that of rGO SC (30%) as the temperature increases from 30 to 90 °C. The observation reveals that the addition of CB being a heat-resistant additive helps improve performance of thermal stable SCs.

## Introduction

In recent, supercapacitors (SCs) have received attention as one of energy storage devices for portable electronics due to their high power density, fast charge/discharge rates and long cycle lifetimes^1–3^. Moreover, SCs operating in an electrostatic charging mechanism are less vulnerable to changes in their operating environment, compared to lithium-ion batteries operating in the mechanism of chemical intercalation^4–6^. Owing to the relative merits of SCs, they have been regarded as one of promising energy storage devices for not only wireless sensors being foundational elements for progress in IoT communications, but also all-in-one energy harvesting and storage devices combined with energy harvesting systems such as solar cells and thermoelectric generators^7,8^. Until now, there have been some studies on the performance of SCs under environmental conditions; Liu *et al*. developed SCs constructed with the ionogel-mask hybrid electrolyte operating at a high pressure of 3236 kPa and 200 °C^9^, Kotz *et al*. investigated the temperature behaviors of SCs and determined acceleration factors for capacitor degradation^10^, and Masarapu *et al*. studied the temperature effect on specific capacitance of SCs^11^. Nevertheless, there are few studies for the improvement on thermal stability of SCs. The thermal stability of SCs is of paramount importance since the energy storage performances of the SCs depend heavily on the change of temperature. In order to acquire sustainable SCs, it is essential to increase the thermal stability of the SCs because the sustainability in the SCs is deeply concerned with deterioration of the performance resulting from the thermal fatigue. Hence, in this study, we propose a simple way to improve the thermal stability of the SCs by mixing reduced graphene oxide (rGO) electrode material and carbon black (CB) used as a heat-resistant additive. In this study, we investigate the effect of the CB addition on the electrochemical characteristics of the rGO-CB SCs in a temperature range from 30 to 90 °C which corresponds to an operating temperature range of the SCs composed of an aqueous gel electrolyte.

## Results and Discussion

**Figure 1:**
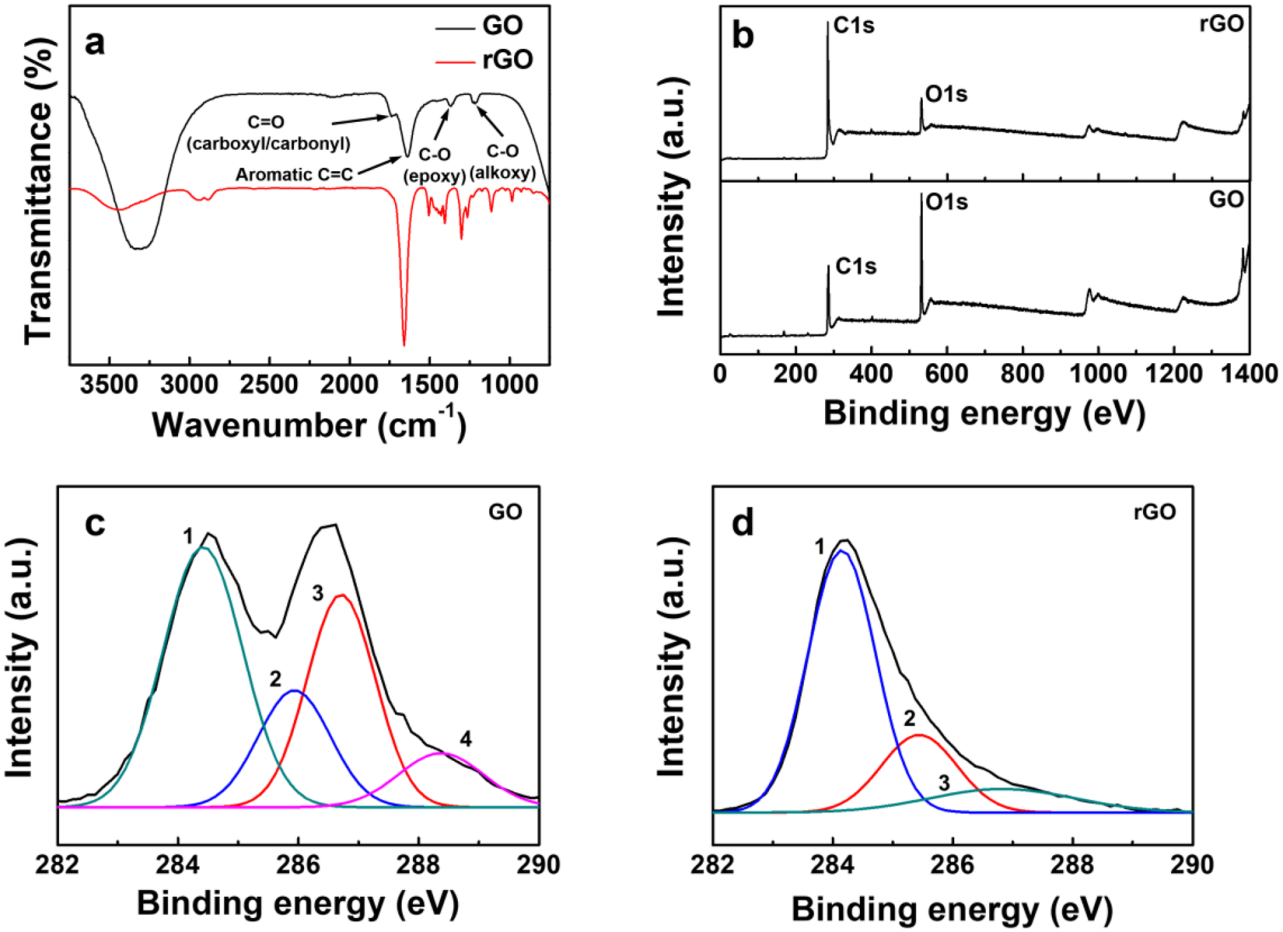
(**a**) FT-IR spectra of the GO and rGO. (**b**) XPS spectra of the GO and the rGO and C1s XPS spectra of (**c**) the GO and (**d**) the rGO.

The reduction from GO to rGO is confirmed by the FT-IR and XPS spectra as shown in Fig. 1. The FT-IR spectra of Fig. 1(a) represent that the intensities of peaks relating to carbon-oxygen bonds shown in GO decrease in rGO; carboxyl/carbonyl stretching (C=O; 1737 cm^−1^), epoxy (C-O; 1368 cm^−1^) and alkoxy (C-O; 1215 cm^−1^) stretching. On the other hand, the intensity of peak corresponding to aromatic C=C stretching (1659 cm^−1^) increases in rGO, which demonstrates the occurrence of reduction from GO to rGO. Figure 1(b) shows the XPS spectra of the GO and the rGO, indicating the peaks associated with C1s and O1s. Compared with the peak intensities of C1s and O1s for the GO and the rGO, the atomic ratio of C1s to O1s increases from 1.8 for the GO to 6.2 for the rGO, which means the deoxygenation of GO by the thermal reduction process. In the C1s XPS spectra of the GO as shown in Fig. 1(c), we found four components to account for the overlapping C1s features: C-C (sp3 C, peak curve 1; 284.5 eV), C-O (epoxy C, peak curve 2; 286.8 eV), C=O (carbonyl C, peak curve 3; 287.8 eV) and -COOH (carboxyl C, peak curve 4; 289.0 eV)^12^. On the other hand, low-intensity peaks related with carbon-oxygen bonds appear in the C1s XPS spectra of the rGO as exhibited in Fig. 1(d). In addition, the proportion of the sp^3^ carbon peak increases from 40.5 to 72.1%, indicating that the GO was sufficiently deoxygenated.

**Figure 2:**
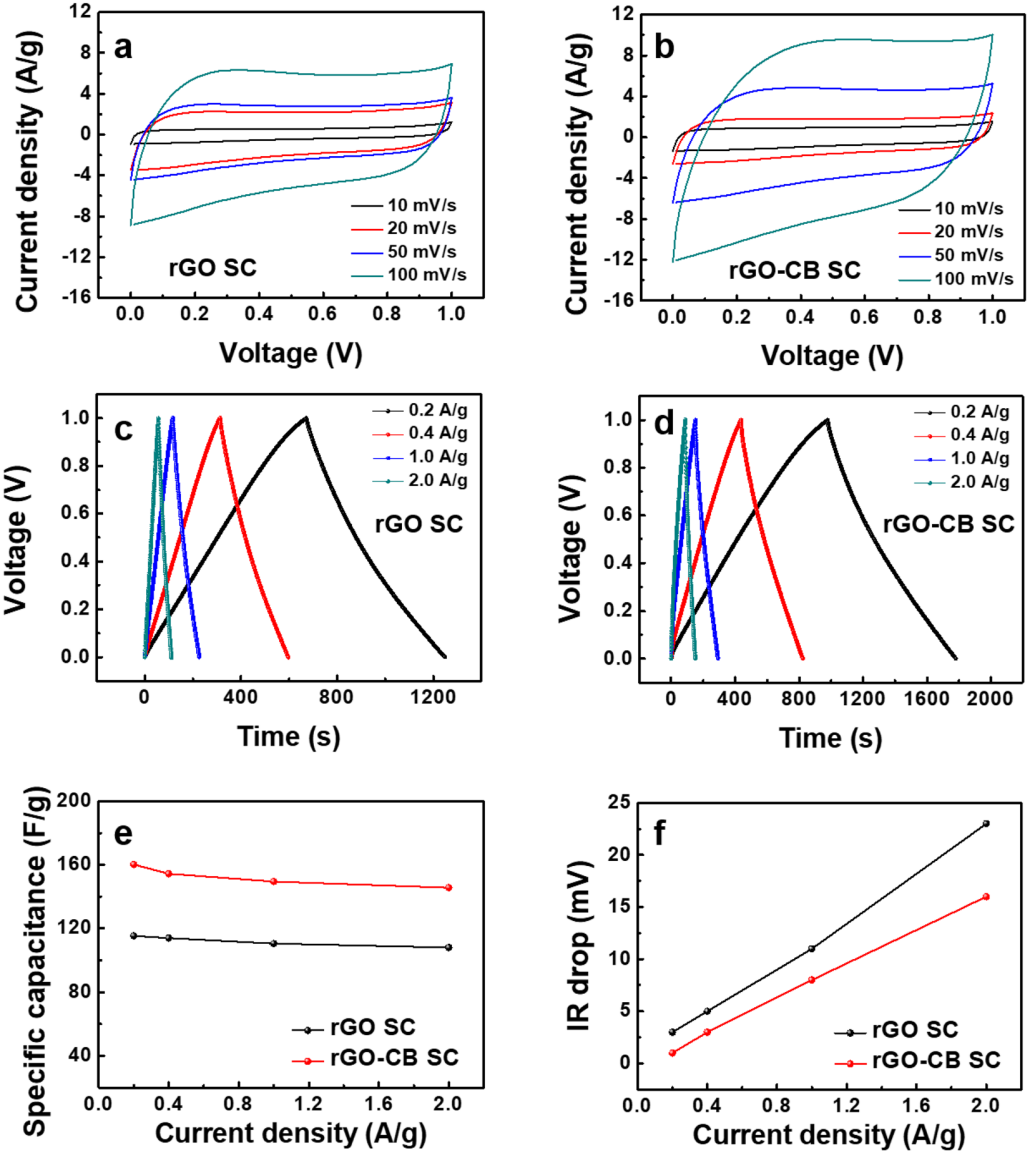
Cyclic voltammetry curves of (**a**) the rGO SC and (**b**) the rGO-CB SC. Galvanostatic charge/discharge curves of (**c**) the rGO SC and (**d**) the rGO-CB SC. Specific capacitance (**e**) plots and *IR* drops (**f**) of the rGO SC and the rGO-CB SC at various current densities.

Figure 2 shows the electrochemical performance of the rGO SC and the rGO-CB SC. The CV analysis of the rGO SC and rGO-CB SC were conducted in a voltage range of 0 to 1.0 V at scan rates of 10, 20, 50, and 100 mV/s, as shown in Fig. 2(a,b). The CV curves of the rGO-CB SC show the larger areas and more quasi-rectangular shapes with smaller distortions for four different scan rates than those of the rGO SC since the added CB improves the conductivity of the electrodes. Considering that the relationship between the electrical properties of SCs and the electrical conductivity of electrodes^13,14^, the rGO-CB SC has the better capacitive behavior and higher mobility of charge carriers in the electrodes, compared to the rGO SC. Figure 2(c,d) exhibit the GCD curves of the rGO SC and rGO-CB SC, respectively, and all curves show the symmetric triangular shapes indicating a high faradaic efficiency during electrochemical reactions^15^. The specific capacitance is calculated by the equation C = (*I*/*m*)·Δt/ΔV, where *I* is the applied current, *m* is the total mass of active electrode materials, Δt is the discharge time, and ΔV is the cell voltage after subtracting the *IR* drop from discharging voltage^16^. Herein, the *IR* drop means an instantaneous voltage drop at the beginning of the discharging state and is attributed to the equivalent series resistance (ESR) that is combined by the resistances of the electrolyte, the active electrode materials, the current collectors and the contacts of the SC^17^. Figure 2(e) exhibits the specific capacitances of the rGO SC and the rGO-CB SC, and at a current density of 0.2 A/g, the specific capacitances of rGO SC and rGO-CB SC are 115 F/g and 160 F/g, respectively. As a current density increases, the specific capacitances of both SCs slightly decrease. Nevertheless, the rGO-CB SC shows 40% larger specific capacitance than the rGO SC. Figure 2(f) represents the *IR* drops of the rGO and the rGO-CB SCs, indicating that the IR drops of the rGO-CB SC are smaller than those of the rGO SC in all cases.

**Figure 3:**
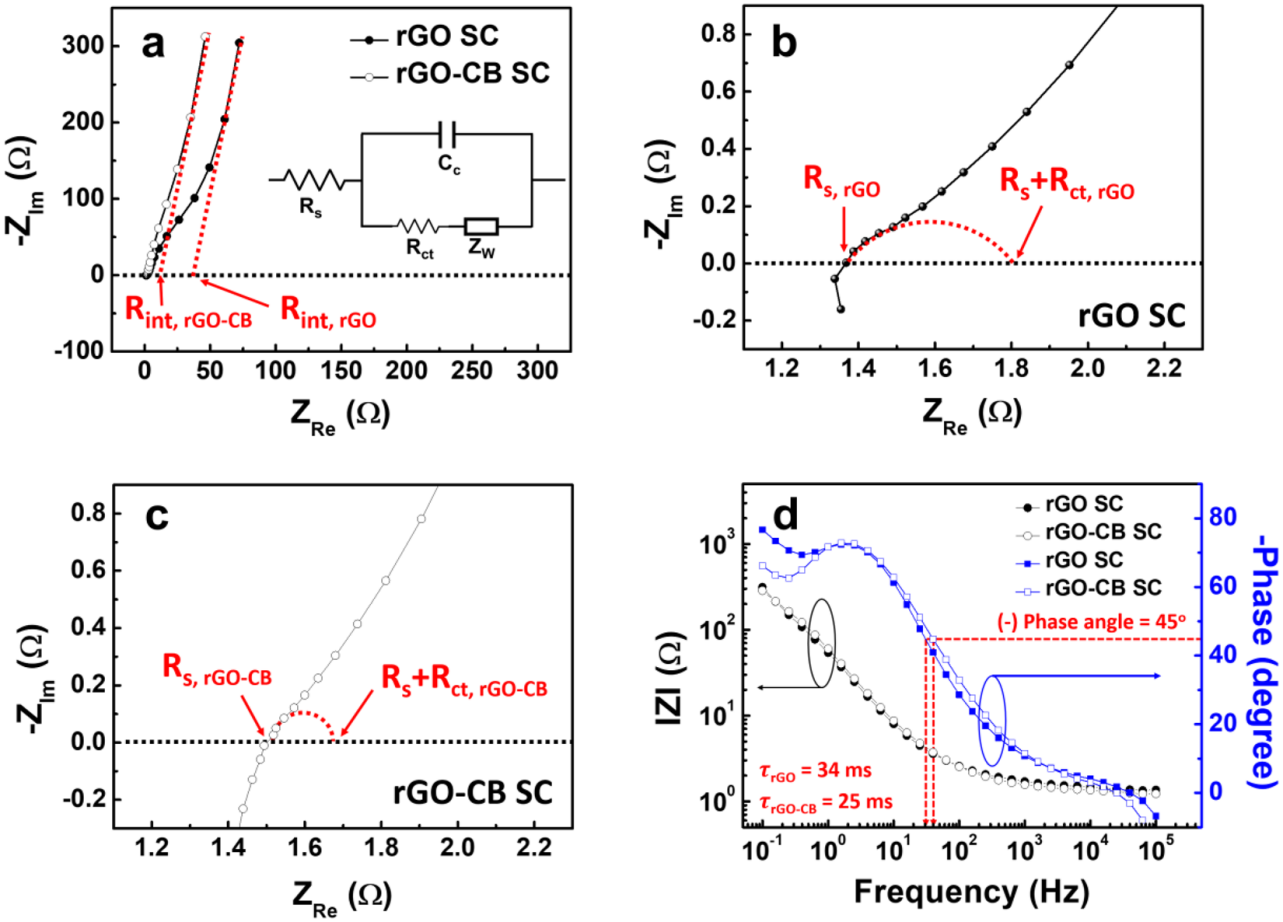
(**a**–**c**) Nyquist plots of the rGO SC and rGO-CB SC for a frequency range from 0.1 MHz to 0.1 Hz, and (**d**) Bode plot of the rGO SC and rGO-CB SC.

The effects of CB on the ESR of SCs are examined by the impedance spectroscopy analysis. Figure 3 shows the Nyquist plots (a) of the rGO SC and the rGO-CB SC in a frequency range from 0.1 Hz to 0.1 MHz and the magnified Nyquist plots (b, c) in a higher frequency region; in the Nyquist plots, the Z′ axis and the Z″ axis are the real and imaginary parts of complex impedance, respectively. The Nyquist plots of both SCs match up with Randle’s equivalent circuit (in the inset of Fig. 3(a)) and consist of semicircles, diffusion lines and capacitive lines. In the semicircle of Nyquist plot, the left intersection on the Z′ axis indicates electrolyte resistance (*R*_s_) and the diameter of the semicircle represents the charge transfer resistance (*R*_*ct*_) including both the electronic and ionic resistances. As shown in Fig. 3(b,c), there is no significant difference between the *R*_*s*_ values of the rGO SC and the rGO-CB SC because the same electrolyte and separator were used for both the rGO SC and the rGO-CB SC. The electronic resistance depends on the electrical conductivity of electrode materials (rGO or rGO-CB) and the electrical contact at the interface between the electrode material and the current collector. In the low frequency region, the x-intercept of the Nyquist plot indicates the internal resistance (*R*_*int*_) of the SC^18^. Compared with the rGO SC (*R*_*ct*_ = 0.4 Ω and *R*_*int*_ = 42 Ω), the rGO-CB SC has relatively low values of *R*_*ct*_ (0.2 Ω) and *R*_*int*_ (18 Ω), indicating that the addition of conductive CB improves electrical contacts between rGO sheets and as a result, lowers the internal resistance of the SC. According to the previous study^19^, the conductive CB provides good electrical connects between rGO sheets since the CB serves as a linker between rGO sheets, which accords with our results. Figure 3(d) exhibits the Bode plots of the rGO SC and rGO-CB SC. The red dash lines indicate the knee frequency where the (−) phase angle reaches 45° and the resistance and the reactance have the same magnitude at that point. From the reciprocal of the knee frequency, the relaxation time constants (τ) of the rGO SC and rGO-CB SC are calculated to be 34 and 25 ms, respectively. Low τ indicates the fast frequency response capability. Considering that the frequency response capability is related to the internal resistance and the electrical conductivity of the SC electrodes^20^, it is clear that the addition of CB lowers the internal resistance and improves the electrical conductivity of SC electrodes. The Randle’s circuit elements and τ of the rGO SC and rGO-CB SC are summarized in Table 1.

**Table 1:** Randle’s equivalent circuit elements of rGO SC and rGO-CB SC.

	R_s_ (Ω)	R_ct_ (Ω)	R_int_ (Ω)	*τ* (ms)
rGO SC	1.4	0.4	42	34
rGO-CB SC	1.5	0.2	18	25

**Figure 4:**
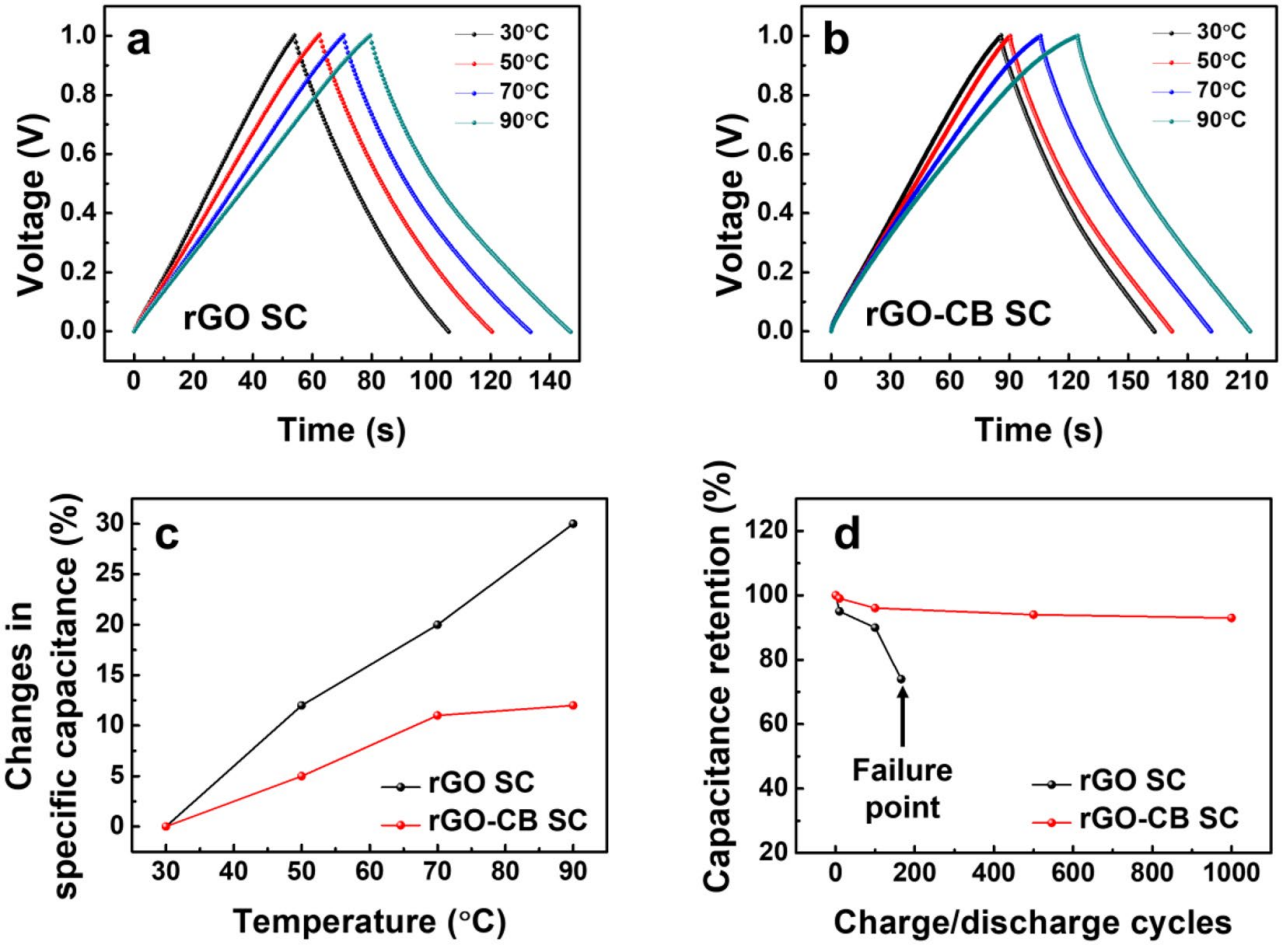
Galvanostatic curves of (**a**) the rGO SC and (**b**) the rGO-CB SC as a function of temperature. (**c**) Variations in specific capacitance of the rGO SC and the rGO-CB SC as a function of temperature. (**d**) Capacitance retention test of rGO SC and rGO-CB SC for 1000 charge/discharge cycles at 90 °C.

In order to investigate the effect of CB on thermal stability of SCs, the GCD curves of the rGO SC and rGO-CB SC are obtained in a temperature range from 30 to 90 °C at a current density of 2 A/g, as shown in Fig. 4(a,b). As the temperature increases, the rGO SC shows larger variations in charging/discharging times than the rGO-CB SC, which is directly related to the variations in specific capacitances of the rGO SC and the rGO-CB SC. Figure 4(c) represents the variations in specific capacitances of the rGO SC and the rGO-CB SC as a function of temperature. The rGO-CB SC shows a smaller variation in specific capacitance (12%) than that of rGO SC (30%) as the temperature increases from 30 to 90 °C. For the rGO-CB SC, the smaller variation in the specific capacitance originates from the lowering of the thermal conductivity of the electrodes and thereby from the reduction of the amount of heat transferred to the electrolyte; note that the thermal conductivity of CB (0.02 W/m∙K) is lower than that of rGO (0.14~2.87 W/m∙K) and that the added CB induces the phonon scattering on the interface in contact with the rGO sheets^21,22^. Consequently, the thermal stability of the rGO-CB SC is superior to that of the rGO SC. The beneficial effect of the addition of CB into the electrodes is attested by capacitance retention test done at 90 °C. The rGO-CB SC exhibits the good capacitance retention performance during 1000 charge/discharge cycles, whereas the function of the rGO SC fails after the 166th charge/discharge cycle as shown in Fig. 4(d).

**Figure 5:**
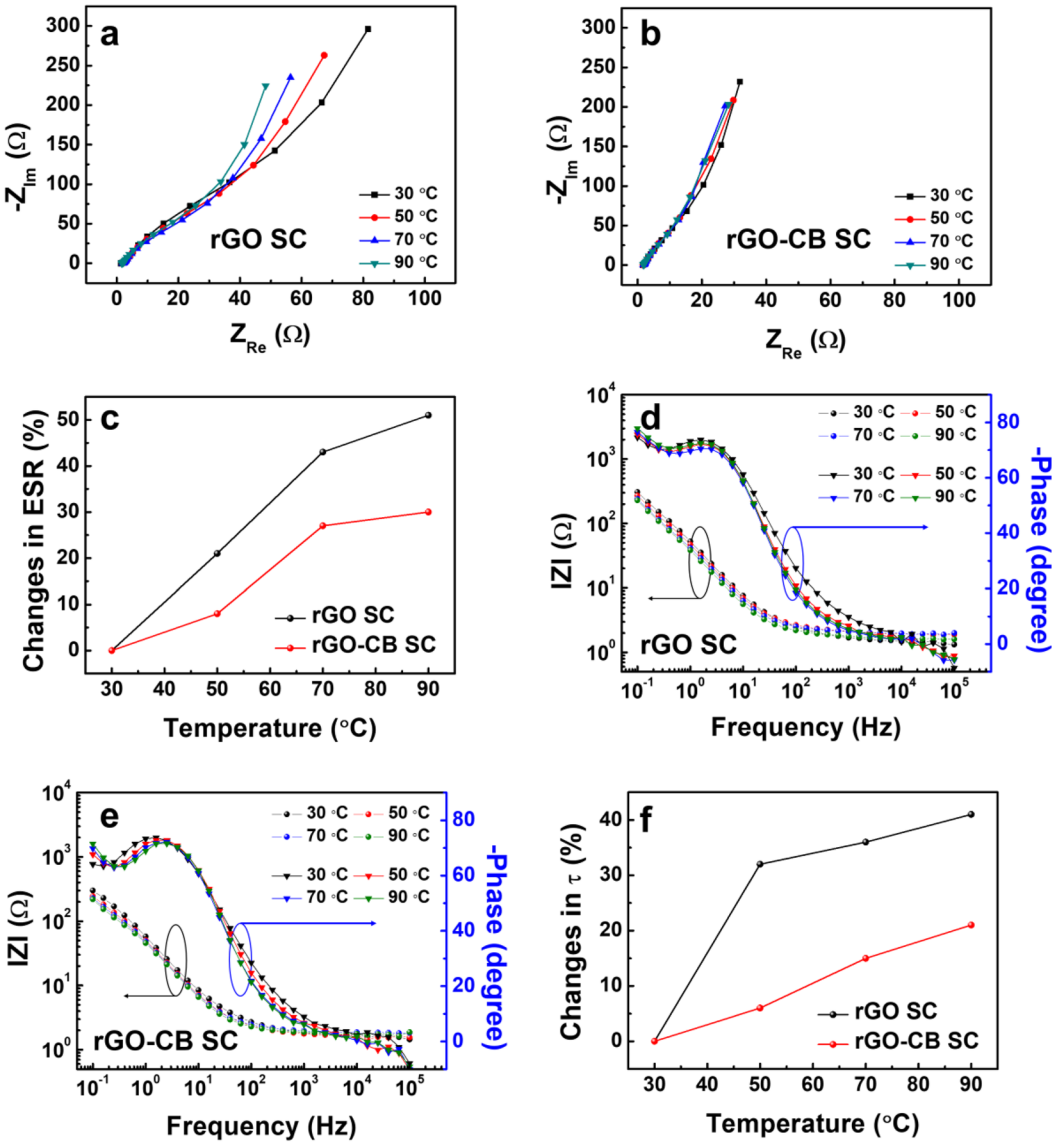
Nyquist plots of (**a**) the rGO SC and (**b**) the rGO-CB SC, and (**c**) the changes in ESR as a function of temperature. Bode plots of (**d**) the rGO SC and (**e**) the rGO-CB SC, and (**f**) the changes in relaxation time constant as a function of temperature.

Furthermore, the effect of CB on thermal stability of SCs is examined with the impedance spectroscopy. Figure 5 show the Nyquist plots of the rGO SC and the rGO-CB SC in a 0.1 Hz to 0.1 MHz frequency range at temperatures of 30~90 °C. As the temperature increases, the curves in the Nyquist plots of the rGO SC and the rGO-CB SC are shifted to the higher frequency region in the *Z*’ direction because of the decrease in the ESR of the rGO SC and the rGO-CB SC shown in Fig. 5(c). As the temperature increases up to 90 °C, the variations in ESR of the rGO SC and the rGO-CB SC are 51% and 30%, respectively. This reveals that the addition of CB with relatively lower thermal conductivity and relatively higher electrical conductivity into the rGO electrodes improves the thermal stability and capacitive performances of SCs. To further investigate the changes in the charge transfer characteristics, we analyzed the Bode plots of the rGO SC and rGO-CB SC as shown in Fig. 5(d,e). As temperature increases from 30 to 90 °C, the phase versus frequency curves of the rGO SC and rGO-CB SC are shifted toward the lower frequency region, and the knee frequency values of both SCs decrease. The amount of the change in τ of the rGO SC and rGO-CB SC, derived from the reciprocal of the knee frequency, are plotted in Fig. 5(f), indicating that the changes in τ of the rGO SC and rGO-CB SC increase up to 21% and 40% as the temperature increase from 30 to 90 °C. Owing to the temperature-dependent charge transfer characteristics of the SCs, the addition of CB being a heat-resistant additive helps improve the performance of thermal stable SCs.

## Conclusion

In this study, we fabricated the solid-state SCs constructed with rGO-CB electrodes and investigated their electrochemical performances and thermal stability. The specific capacitances of rGO SC and rGO-CB SC are 115 F/g and 160 F/g at a current density of 0.2 A/g and the *IR* drops of rGO-CB SC are smaller than those of rGO SC in all cases. In addition, the rGO-CB SC shows a smaller variation in specific capacitance (12%) than that of rGO SC (30%) as the temperature increases from 30 to 90 °C. The rGO-CB SC operates stably even after 1000 charge/discharge cycles at 90 °C, while the rGO SC fails at the 166th cycle. The CB added into the rGO electrode plays roles as the thermal resistor as well as the electrical conductor in the electrodes. From our results, it demonstrates that the addition of CB being a heat-resistant additive helps improve performance of thermal stable SCs.

## Materials and Methods

A GO dispersion in H_2_O (4 mg/mL) and CB powders were purchased from Sigma-Aldrich and MTI corporation, respectively. The GO dispersion was mixed with N-methylpyrrolidone at a volume ratio of 1:7 and the mixture was refluxed at 250 °C for 12 hours, then the thermally reduced GO (rGO) dispersion was prepared. The converting from GO to rGO was confirmed by x-ray photoelectron spectroscopy (XPS; AXIS ULTRADLD spectrophotometer) and FTIR spectroscopy (HORIBA, LabRam ARAMIS IR2 spectrometer). In order to make rGO-CB dispersion, CB powders were dispersed in a refluxed solution with a concentration of 10 wt%. The concentration of CB added to the rGO dispersion was determined through comparing the performances of other SCs with various concentration of CB as shown in Fig. S1 of supplementary information. The existence of CB added to the rGO was confirmed by SEM images as represented in Fig. S2 of supplementary information. On the other hand, Au current collectors were thermally deposited with a thickness of 100 nm on polyethylene terephthalate substrates and, in order to fabricate rGO-CB electrodes, the rGO-CB dispersion was dropped on the Au current collectors and dried in air. To prepare the gel electrolyte used in this study, 0.53 ml of H_3_PO_4_ solution was mixed with 10 ml of PVA aqueous solution (0.1 g/ml; MW_PVA_: 85,000~124,000) and then the mixture was stirred at 70 °C for 10 min. The H_3_PO_4_/PVA gel electrolyte films were made on the rGO-CB electrodes by a dropping method and finally, the rGO-CB SC was completed by overlapping two rGO-CB electrodes coated with the H_3_PO_4_/PVA gel electrolyte films. Additionally, the reference SC with electrodes made of only rGO was fabricated to investigate the effect of CB on performance of the SC. Cyclic voltammetry (CV), galvanostatic charge/discharge (GCD), and impedance analysis of the SCs were carried out with an IviumStat electrochemical workstation. To investigate the thermal stability characteristics of the SCs with aqueous gel electrolytes, the GCD experiments and the impedance analysis of the rGO SC and the rGO-CB SC were performed on a hot plate in a temperature range from 30 to 90 °C. The uniformity of temperature over all surfaces of the SC was confirmed through IR camera images as shown in supplementary information (Fig. S3).

## Electronic supplementary material

Supplementary information
