# Supplementary material for: Effect of carbon black addition on thermal stability and capacitive performances of supercapacitors
Source: Sci Rep. 2018 Aug 10;8:11989. doi: 10.1038/s41598-018-30507-5 (PMC6086910; doi:10.1038/s41598-018-30507-5)
Supplement: Supplementary file 1 — Supplementary information [file 41598_2018_30507_MOESM1_ESM.pdf]

Supplementary information for

Effect of carbon black addition on thermal stability and capacitive performances of supercapacitors

Kyungwhan Yang, Kyoungah Cho*, Sangsig Kim**

Department of Electrical Engineering, Korea University, 146, Anam-ro, Sungbuk-gu, Seoul 02841, Korea

1. Concentration optimization of carbon black (CB) added to reduced graphene oxide (rGO)

In this study, we optimized the concentration of CB added to the rGO electrodes as shown in Fig. S1. Compared to other SCs, the rGO-CB SC with a CB concentration of 10 wt% shows the largest area in the CV curves, the long discharging time in the GCD curves and the largest specific capacitance of 160 F/g.

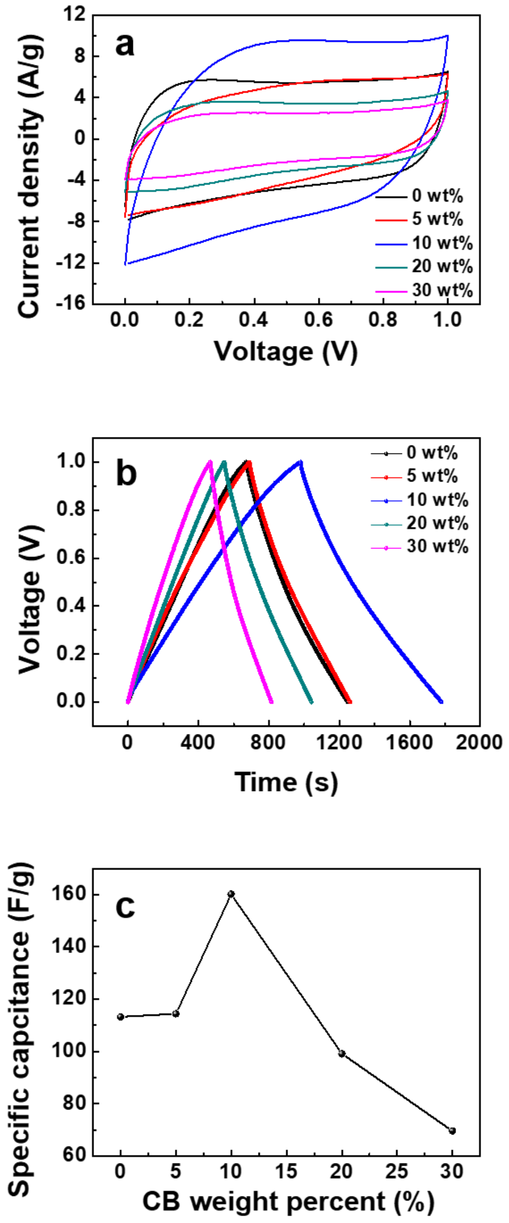

Figure S1. Cyclic voltammetry curves (a) and galvanostatic charge/discharge curves (b) of the rGO-CB SCs constructed with various concentration of CB added to the rGO. (c) Specific capacitance as a function of CB weight percent.

2. Morphologies of the rGO and rGO-CB films

We observed the morphologies of the rGO and rGO-CB from SEM images as shown in Fig. S2. The surface of the rGO film is smooth but the particles of CB appear in surface of the rGO-CB film.

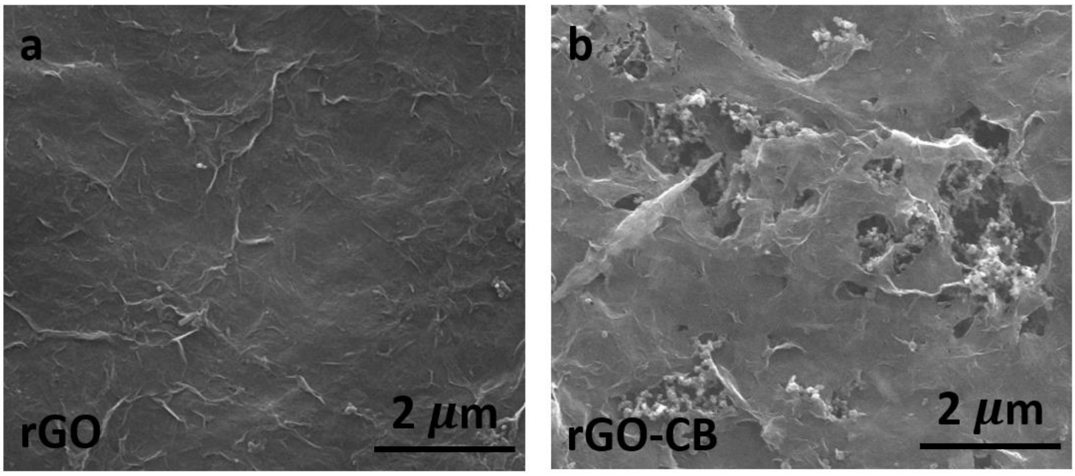

Figure S2. SEM images of the rGO (a) and rGO-CB (b).

3. Temperature uniformity on the surfaces of the rGO SC and rGO-CB SC using IR image analysis

In this study, the temperature distribution of the rGO SC and rGO-CB SC was analyzed using IR images. As shown in Fig. S3(a), the rGO SC and the rGO-CB SC were placed on the hot plate and temperature of the hot plate was changed from 30 to 90 °C. As the set temperature increases from 30 to 90 °C, the surface temperatures uniformly increase for both rGO SC and rGO-CB SC. Nevertheless, the temperature difference between the rGO SC and rGO-CB SC changes from 0.2 to 3.6 °C as shown in Fig. S3(f). This phenomenon demonstrates that CB added to the SC electrodes reduces heat transfer from electrode to electrolyte.

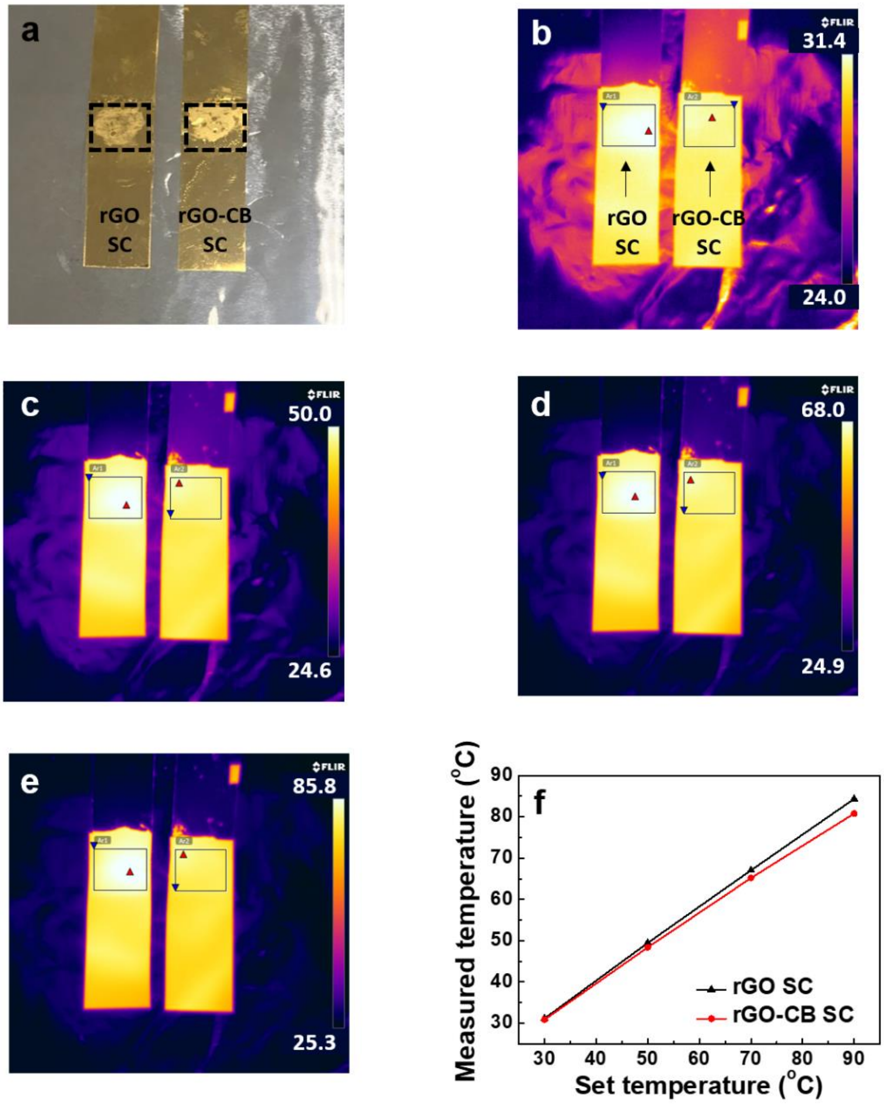

Figure S3. (a) Optical image of the rGO SC and rGO-CB SC on the hot plate. (b-e) IR images of the rGO SC and rGO-CB SC under temperature variation from 30 to 90 °C. (f) Average temperatures of the rGO SC and rGO-CB SC measured by IR images.
